# Pathogenesis and Novel Treatment from the Mouse Model of Type 2 Diabetic Nephropathy

**DOI:** 10.1155/2013/928197

**Published:** 2013-04-24

**Authors:** Masako Furukawa, Tomohito Gohda, Mitsuo Tanimoto, Yasuhiko Tomino

**Affiliations:** Division of Nephrology, Department of Internal Medicine, Juntendo University Faculty of Medicine, 2-1-1 Hongo, Bunkyo-ku, Tokyo 113-8421, Japan

## Abstract

Diabetic nephropathy (DN) is the leading cause of end-stage kidney disease worldwide. However, current treatments remain suboptimal. Many factors, such as genetic and nongenetic promoters, hypertension, hyperglycemia, the accumulation of advanced glycation end products (AGEs), dyslipidemia, and albuminuria/proteinuria itself, influence the progression of this disease. It is important to determine the molecular mechanisms and treatment of this disease. The development of diabetes results in the formation of AGEs, oxidative stress, and the activation of the renin-angiotensin-aldosterone system (RAAS) within the kidney, which promotes progressive inflammation and fibrosis, leading to DN and declining renal function. A number of novel therapies have also been tested in the experimental diabetic model, including exercise, inhibitors of the RAAS (angiotensin type 1 receptor blockers (ARB), angiotensin-converting enzyme (ACE) inhibitors), inhibitors of AGE (pyridoxamine), peroxisome proliferator-activated receptor (PPAR) **γ** agonists (pioglitazone), inhibitors of lipid accumulation (statins and eicosapentaenoic acid (EPA)), and the vitamin D analogues. This review summarizes the advances in knowledge gained from our studies and therapeutic interventions that may prevent this disease.

## 1. Introduction

Diabetes mellitus is a major cause of chronic kidney disease (CKD) worldwide [1] and is associated with enhanced morbidity and mortality, particularly with accelerated cardiovascular disease [2].

Current approaches to the prevention of diabetic nephropathy (DN) include the strict control of blood glucose and blood pressure. The strict control of blood glucose, as quickly as possible, was shown to be effective in major clinical trials [3, 4]. Blood pressure control has also been shown to be of major importance in many studies [5–7]. In addition, in patients who develop increased urinary albumin-creatinine ratio (ACR) levels, one or more of the medications that inhibit the renin-angiotensin-aldosterone system (RAAS) axis should be used to lower ACR levels [8, 9]. These treatments remain suboptimal, however, so much more research is needed to determine other specific pathophysiologic mechanisms in order to develop more treatments that are targeted specifically to identified mechanisms.

**Figure 1:**
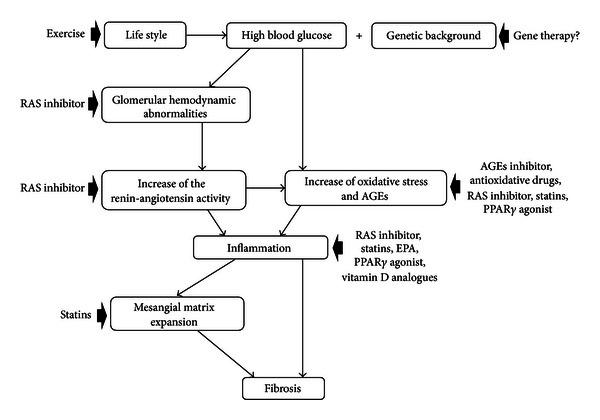
Major mechanistic pathways of diabetic renal injury identified. Various factors contribute to the progression of diabetic nephropathy, as shown in this slide, and there is crosstalk between these factors.

The pathogenesis of DN appears to be multifactorial. Several genetic and environmental factors likely contribute to its development and progression [10]. Diabetes induces the formation of advanced glycation end products (AGEs), which can alter the function of proteins and stimulate pathological cellular responses via AGE receptors. Increasing levels of AGEs, and their deposition in diabetic kidneys, correlate with the development of DN [11]. Of the pathophysiologic mechanisms that have been identified in the development and progression of DN, oxidative stress (more accurately described as increased levels of reactive oxygen species; ROS) is of major importance [12]. Recent studies have shown that kidney inflammation is crucial in promoting the development and progression of DN. Inflammation, which is activated by the metabolic, biochemical, and hemodynamic derangements known to exist in the diabetic kidney, may be a key factor [13]. DN progresses in stages, starting with the thickening of the glomerular basement membrane, mesangial cell expansion, and then gradually progressing into glomerulosclerosis and interstitial fibrosis eventually resulting in renal failure [14]. It has been postulated that the relationship between AGE effects, oxidative stress, RAAS activation, inflammation, and fibrosis pathways plays an important role in the development and progression of DN (Figure 1).

This review focuses on potential targets for new renoprotective therapies from our data in addition to the inhibition of the RAAS in DN.

## 2. Characteristics of KK and KK-A^**y**^ Mouse

The KK mouse is an inbred mouse strain established from Japanese native mice. This mouse spontaneously exhibits type 2 diabetes, associated with mild hyperglycemia, mild glucose intolerance, mild hyperinsulinemia, mild obesity, and mild microalbuminuria. Renal lesions in KK mice closely resemble those in human diabetic nephropathy. Young KK mice are considered to be suitable model for the study of the early stage of type 2 DN in humans [15].

The KK-A^y^ mouse line was established in 1969, and these mice are widely used as an experimental model for type 2 diabetes mellitus. KK-A^y^ mice spontaneously exhibit type 2 diabetes mellitus signs, including hyperglycemia, glucose intolerance, hyperinsulinemia, obesity, and microalbuminuria. The mice also develop renal lesions that show diffuse hyperplasia of the mesangial area with mesangial cell (MC) proliferation, segmental sclerosis, overexpression of TGF-*β*1, and the accumulation of AGEs and ROS products [16–18].

## 3. Treatment of Diabetic Nephropathy

### 3.1. The Effect of Renin-Angiotensin-Aldosterone System Inhibitors

 Angiotensin II (Ang II) exerts both hemodynamic effects, leading to increased glomerular capillary pressure, and nonhemodynamic effects such as cellular hypertrophy stimulation and extracellular matrix (ECM) accumulation. These effects are mediated through the interaction of Ang II with its angiotensin type 1 (AT1) receptor. Angiotensin-converting enzyme (ACE) inhibitors and AT1 receptor blockers (ARB) have been demonstrated to improve glomerular hemodynamics and structures in both human and experimental DN [19–21]. Our study demonstrated that treatment with candesartan, an ARB, reduced blood pressure and mesangial ECM accumulation and reduced ACR and type IV collagen excretion without altering glucose metabolism [22]. Many studies have reported that high glucose and Ang II stimulate collagen production by TGF-*β* activation [23, 24]. TGF-*β* is an important mediator of fibrosis in the repair tissues. Smad7 is generally considered to be a TGF-*β* signaling inhibitor in mature T cells. In our study, TGF-*β* expression by immunohistochemistry in glomeruli was markedly increased in the mild diabetic model of KK mice. Candesartan administration significantly reduced TGF-*β* expression. Our data also demonstrated that candesartan treatment led to an increase in glomerular Smad7 expression [22]. It appears that these protective effects of candesartan were associated with lower glomerular hydraulic pressure, reduced TGF-*β* expression, and increased Smad7 expression. These data supported the previous data that the TGF-*β*/Smad signal system could play an important role in the development and progression of DN in KK mice [15].

 Oxidative and nitrosative stresses are widely recognized as key factors in the development of DN. We demonstrated that nitrooxidative stress and AGE production are enhanced in the kidneys of KK mice. Candesartan decreased nitrooxidative stress by downregulating NAD(P)H oxidase p37phox and iNOS expression, and modified interaction between AGEs and RAGE by attenuating RAGE expression, contributing to the reduction of AGE accumulation and subsequent albuminuria [15, 25].

 Evidence has accumulated over the past few years indicating that adenosine monophosphate activated protein kinase (AMPK) may be a useful target for the pharmacologic treatment of type 2 diabetes. The actions of AMPK were initially defined as the regulation of fatty acid and cholesterol synthesis pathways [26]. In parallel with their activation of AMPK, antidiabetic adipokines, that is, adiponectin, stimulate phosphorylation of acetyl CoA carboxylase (ACC), fatty acid oxidation, glucose uptake, and lactate production. Our data demonstrated that enalapril and/or losartan improved the urinary ACR levels through the activation of adiponectin and AMPK in the kidneys of KK-A^y^ mice [27]. These results suggested that the RAS inhibitors activated renal AMPK through its phosphorylation. Therefore, the effects of ACE inhibitors and/or ARBs, especially in combination treatment, might be associated with tissue-specific adiponectin-AMPK activity.

### 3.2. Effect of Angiotensin-(1–7)

There is emerging evidence that in DN, the generation of ROS is a major factor in the development of diabetes and its associated complications [28, 29]. NAD(P)H oxidase is an enzymatic complex that is responsible for ROS production. Ang II-mediated ROS is an important second messenger for the transcriptional effects of Ang II, and NAD(P)H oxidase is the central enzyme complex of Ang II-induced ROS [30, 31]. The recent discovery of the renal RAS [28, 29], ACE carboxypeptidase (ACE2), and Ang-(1–7) has changed the way the RAS is viewed. Ang-(1–7) is a biologically active heptapeptide component of the RAS and is generated in the kidney at relatively high levels via enzymatic pathways that include ACE2. The biological effects of Ang-(1–7) in the kidney are primarily mediated by their interaction with the G-protein-coupled receptor Mas [32]. We found that mice coinfused with Ang II + Ang-(1–7) had a lower increase in urinary ACR than mice infused with Ang II alone. In this animal model, Ang-(1–7) attenuated Ang II-mediated NAD(P)H oxidase activation and ROS production in diabetic glomeruli and MCs. These findings were related to improved mesangial expansion and the production of fibronectin and TGF-*β*1 in the diabetic kidney and cultured MCs as opposed to Ang II. We also found that Ang II-induced NF-*κ*B and MAPK activation was attenuated by Ang-(1–7) in the MCs [18]. Our findings suggest that Ang-(1–7) may attenuate Ang II-stimulated NAD(P)H dependent ROS-mediated renal injury in diabetes. The ACE2-Ang-(1–7)-Mas receptor axis should be investigated further as a novel target of treatment of DN.

### 3.3. Effect of Pyridoxamine

Chronic hyperglycemia promotes the generation of AGEs as a result of sequential biochemical reactions involving the nonenzymatic glycation of protein and lipids [33]. The formation of AGEs occurs in normal aging, but it is accelerated in the diabetic state. AGEs increase the expression of growth factors and cytokines, including TGF-*β*, connective tissue growth factor (CTGF), and vascular endothelial growth factor (VEGF) [34]. AGEs can induce the expression of the monocyte chemoattractant protein-1 (MCP-1) in podocytes through the activation of the AGE receptor (RAGE) and the generation of intracellular ROS [35]. Eventually, these products cause glomerular and tubulointerstitial injury. In therapeutic interventions for reducing AGEs, many compounds have been reported as AGE inhibitors, such as aminoguanidine, phenacylthiazolium bromide, 2-isopropylidenehydrazono-4-oxo-thiazolidine-5-yl-acetanilide (OPB-9195), 2,3-diaminophenazine, vitamin C, vitamin E, Ang II receptor inhibitor, and pyridoxamine [25, 36–38]. Pyridoxamine was introduced by Khalifah et al. [39, 40] as an inhibitor of AGE formation from Amadori products [41]. The effects of pyridoxamine include (a) the inhibition of AGE formation by blocking the oxidative degradation of the Amadori intermediate of the Millard reaction, (b) the scavenging of toxic carbonyl products of glucose and lipid degradation, and (c) the trapping of ROS [42]. We demonstrated that pyridoxamine (K-163) ameliorates the levels of urinary ACR and serum 3-deoxyglucosone (3DG) in KK-A^y^ mice without changing systemic blood pressure. Furthermore, pyridoxamine prevented accumulations of Nq-(carboxymethyl)lysine (CML), nitrotyrosine, TGF-*β*1, and laminin-*β*1 in the kidney tissues [41]. AGEs and oxidative stress might activate autocrine Ang II signaling and subsequently induce TGF-*β*1-Smad signaling in mesangial cells [43, 44]. Our findings suggested that the amelioration of urinary ACR was related to the improvement of TGF-*β*1 and laminin-*β*1 expressions in the kidney because CML and nitrotyrosine accumulations were improved and the levels of serum 3DG were reduced by anti-AGE and/or the antioxidant effects of pyridoxamine.

### 3.4. Effect of Pioglitazone

Thiazolidinediones (TZDs), PPAR*γ* agonists, such as troglitazone, pioglitazone, and rosiglitazone, are insulin-sensitizing agents [45]. It is generally considered that these drugs have preventive effects on impaired glucose tolerance (IGT) and urinary ACR excretion in diabetics [46–48]. In the kidney, PPAR*γ* messages were localized predominantly in the inner medullary collecting ducts and renal medullary interstitial cells but not in the cortex [49]. However, previous reports have shown that TZDs ameliorate renal microcirculation, glomerular hyperfiltration, and mesangial expansion in DN [50–52]. The increase of renal perfusion and glomerular filtration rate (GFR) occurs early in the course of DN. Nitric oxide (NO) might be one of the causes of glomerular hyperfiltration [53]. Veelken et al. [54] reported that early glomerular hyperfiltration was dependent on increased NO generation due to greater expression and activity of endothelial constitutive NOS (ecNOS) in glomeruli and afferent arterioles in untreated hyperfiltrating diabetic rats. Therefore, ecNOS might be a more important hemodynamic factor in the early stage of DN. We demonstrated that pioglitazone, one of the TZDs, ameliorates urinary ACR and IGT in diabetic KK mice without changing systemic blood pressure and blood glucose levels. We localized ecNOS protein in the endothelium of preglomerular arteries, arterioles, and glomerular tufts. Moreover, this positive staining in KK mice treated with pioglitazone was less than that in control mice. It appears that the decrease of urinary ACR excretion might be related to the improvement of glomerular enlargement, including hyperfiltration, since the levels of ecNOS protein were reduced by pioglitazone in the glomerular vessels [55].

### 3.5. Effect of Eicosapentaenoic Acid

Eicosapentaenoic acid (EPA) is one of the n-3 polyunsaturated fatty acids (PUFA) which are contained in fish oil. It was shown that EPA has many effects, such as antithrombotic, hypolipidemic, antiatherogenic, anti-inflammatory, and antimitogenic actions. The feeding of fish oil rich in n-3 PUFA reduces the ex vivo production of interleukin-1 (IL-1), IL-6, tumour necrosis factor (TNF), and IL-2 by peripheral blood mononuclear cells and reduces the response to endotoxin and to proinflammatory cytokines, resulting in increased survival. MCP-1 is the strongest known chemokine, which has the function of recruiting and activating monocytes/macrophages from the circulation to inflammatory sites. Macrophages and its products play an important pathogenic role in the tubulointerstitial inflammatory and noninflammatory conditions and have been implicated as effector cells of tubulointerstitial damage and mesangial matrix accumulation in DN [56]. We demonstrated that EPA, one of the n-3PUFA, ameliorates urinary ACR and MCP-1 levels and attenuates mesangial matrix accumulation and tubulointerstitial fibrosis in KK-A^y^ mice without changing systemic blood pressure and fasting blood glucose levels. Moreover, EPA ameliorates IGT and hypertriglyceridemia and lowers leptin levels in KK-A^y^ mice. Because MCP-1 induces monocyte immigration and differentiation to macrophages, which augment extracellular matrix production and tubulointerstitial fibrosis, and also because it directly induces tubulointerstitial inflammation and vascular damage in the kidney, we propose that the observed downregulation of MCP-1 is critically involved in the beneficial effect of EPA, probably in concert with the improvement of other clinical parameters. The potential of EPA in the treatment of DN might be of particular relevance to patients with comorbidities such as dyslipidaemia and obesity [57].

### 3.6. Effect of Statins

The 3-hydroxy-3-methylglutaryl-coenzyme A (HMGCoA) reductase inhibitors (statins) have pleiotropic effects on cardiovascular, cerebrovascular, and microvascular diseases independent of their cholesterol-lowering effect [58, 59]. Statins also have beneficial effects on kidney disease, including DN. Previous reports have shown the pleiotropic effects of statins, such as their anti-inflammatory effects and antioxidative stress effects in vitro and in vivo [58, 60]. Mechanisms of improvement of urinary ACR by statin treatment have been proposed in some reports [59, 61, 62]. These reports have stated that statins improved the urinary ACR of diabetic rats through an anti-inflammatory effect and/or through the inhibition of macrophage recruitment and activation and also by the inhibition of TGF-*β* overexpression. We suggested that oxidative stress and nitrotyrosine are related to the progression of DN [41]. Oxidative stress is defined as a tissue injury induced by an increase of ROS, such as the hydroxyl radical, superoxide anion, or hydrogen peroxide. Thus, oxidative stress is considered to be one of the factors involved in the development of diabetic complications [63, 64]. Isoprenoids, such as farnesyl pyrophosphate or geranylgeranyl pyrophosphate, are generated from HMG-CoA through mevalonate depletion. Isoprenoids inhibit the generation of eNOS and GTPCH-1, and they also increase NAD(P)H oxidase through the inhibition of the Rho pathway and activation of Rac-1. Since pitavastatin inhibits HMGCoA reductase and blocks synthesis of the isoprenoids, the generation of NAD(P)H oxidase is inhibited and signals to generate eNOS are upregulated. Furthermore, statins activate GTPCH-1 and lead to the upregulation of BH4, which is essential for eNOS to form a dimer. As a result, pitavastatin activates eNOS dimerization and enforces their stability through this cascade. As the monomerization of eNOS, which involves NO and ROS imbalance, is decreased, oxidative stress is decreased. Moreover, upregulated dimeric eNOS acts as an NO generator and may work against shear stress in the early stage of DN [17]. We demonstrated that pitavastatin improves the levels of urinary ACR, urinary 8-OHdG, and insulin resistance in KK-A^y^ mice independent of cholesterol-lowering effect. Furthermore, pitavastatin prevented the accumulation of monomeric eNOS, nitrotyrosine, and p47 phox in kidney tissues. It appears that pitavastatin improved not only urinary ACR but also HbA1c and impaired glucose tolerance in KK-A^y^ mice, which might be because of the suppression of eNOS uncoupling and its antioxidant effects on DN.

### 3.7. Effect of Vitamin D Analogues

The natural activator of the vitamin D receptor, calcitriol, is produced by the kidney, but plasma concentrations decline as estimated GFR (eGFR) reduces [65]. In a multivariable analysis of patients with chronic kidney disease (CKD), lower calcitriol concentrations strongly correlated with higher risk of diabetes, higher urinary ACR, and lower eGFR [65]. Calcitriol, 1,25-dihydroxyvitamin D 3 (1,25(OH) 2 D 3), and its analogs have been shown to attenuate renal diseases [66–68]. For example, the knockout of the vitamin D receptor in diabetic mice was associated with severe albuminuria and glomerulosclerosis from increased thickening of the glomerular basement membrane (GBM) and podocyte effacement [69]. 1,25(OH) 2 D 3 is a negative endocrine regulator of RAS and suppresses renin biosynthesis [70, 71]. These studies provide the molecular basis for exploring the potential of 1,25(OH) 2 D 3 to regulate the RAS by inhibition of renin [70]. We investigated the effect of therapy with 1,25(OH) 2 D 3 upon DN in KK-A^y^ mice. It appears that therapy with 1,25(OH) 2 D 3 reduced the urinary ACR level by suppressing the compensatory renin increase in type 2 DN. These beneficial effects might be related to suppressed renal expression of renin, ERK1/2, and TGF-*β* which may or may not be Ang II dependent [72]. Moreover, a recent trial reported antialbuminuric effects of another analogue, paricalcitol, further strengthening the evidence for vitamin D analogues as renoprotective agents [73]. Paricalcitol lowers residual albuminuria in patients with DN and could be a novel approach to lower residual renal risk in diabetes.

### 3.8. Effect of Exercise

Lifestyle modification, especially appropriate exercise, is recommended for the management of type 2 DN through improvements of metabolic risk factors such as blood pressure, blood glucose, plasma lipids, and oxidative stress markers. On the other hand, appropriate exercise also consumes considerable amounts of oxygen, leading to the production of high levels of ROS. There is also evidence that ROS and high glucose exposure contribute to podocyte apoptosis in experimental DN [74]. There are several mechanisms for the renoprotective effects of exercise in DN. In general, exercise training ameliorates renal function by improving metabolic factors such as plasma lipids, blood glucose, blood pressure, and body weight. It is also known to improve renal histology without altering metabolic factors. Boor et al. [75] demonstrated that exercise training reduced AGEs in both serum and kidney tissues of obese Zucker rats, an animal model of type 2 diabetes, without altering inflammatory biomarkers or metabolic factors. In contrast, our study clearly showed that the exercised mice showed attenuated renal expression of MCP-1 and infiltration of macrophage in the kidneys. We demonstrated that the exercise training improved urinary NAG levels as well as the change rate of urinary ACR, independent of body weight and glycemic status in the kidneys of KK-A^y^ mice, although moderate-intensity exercise increased expression of HIF-1*α* in the kidneys. In our study, no significant changes were observed in the levels of Ccr between sedentary KK-A^y^ and exercised KK-A^y^ mice. Therefore, it is indicated that the decrease of urinary ACR was not due to the reduction of renal blood flow/glomerular filtration rate, but more likely to the effect of exercise. It is thought that appropriate exercise increases antioxidant enzymes, although excessive exercise causes inflammation, increases oxidative stress associated with ROS, and decreases the renal blood flow and glomerular filtration rate. In our study, both exercises decreased urinary 8-OHdG levels, an oxidative stress marker. However, contrary to our expectation, low-intensity exercise was more effective than moderate-intensity exercise in terms of renal function. Further investigation is required to determine appropriate exercise intensity. It appears that low-intensity exercise attenuates the progression of early DN without affecting marked renal ischemia. Thus, attention should be paid to renal ischemia even though albuminuria has improved. Reductions in the rate of urinary ACR change, urinary NAG, and maintained podocyte numbers, with parallel improvements in oxidative damage and chronic inflammation, might be related to beneficial effects of exercise in DN [76].

## 4. Conclusion

Despite the successful use of lifestyle changes, metabolic control, and blood pressure control, including ACE inhibitors and ARB therapy, residual renal risk remains very high, leaving the diabetic population with a clear unmet need for novel treatment options. As outlined in this review, various drugs are in development. It is anticipated that some of the newer agents that are currently the focus of clinical trials will ultimately lead to improvements in slowing the progression and eventually improving the prognosis of this devastating disease.
